# IL-6 mediates differentiation disorder during spermatogenesis in obesity-associated inflammation by affecting the expression of Zfp637 through the SOCS3/STAT3 pathway

**DOI:** 10.1038/srep28012

**Published:** 2016-06-22

**Authors:** Guizhen Huang, Miao Yuan, Jie Zhang, Jun Li, Di Gong, Yanyan Li, Jie Zhang, Ping Lin, Lugang Huang

**Affiliations:** 1 Department of Pediatric Surgery, West China Hospital, Sichuan University, Chengdu, China; 2 Division of Experimental Oncology, State Key Laboratory of Biotherapy, West China Hospital, Sichuan University, and Collaborative Innovation Center for Biotherapy, Chengdu, China

## Abstract

Zfp637 is a recently identified zinc finger protein, and its functions remain largely unknown. Here, we innovatively demonstrate the effects of Zfp637 on the differentiation of mouse spermatogonia and on its downstream target gene SOX2 *in vitro*. Obesity has been recognized as a chronic inflammatory disease that leads to decreased sexual function and sexual development disorders. We observed higher levels of IL-6 in serum and testis homogenates from obese mice compared with control mice. We also demonstrated that high levels of IL-6 inhibited Zfp637 expression, and we elucidated the underlying mechanisms. SOCS3 overexpression and STAT3 phosphorylation inhibitor (AG490) were used to investigate the function of the SOCS3/STAT3 pathway during this process. Our results showed that exposure of mouse spermatogonial cells to high levels of IL-6 inhibited Zfp637 expression by increasing SOCS3 expression and inhibiting the phosphorylation of STAT3, further reducing cellular differentiation. Consistent with the *in vitro* results, we observed increasing expression levels of SOCS3 and SOX2, but a reduction of Zfp637 expression, in obese mouse testes. In conclusion, Zfp637 plays a crucial role in spermatogenesis by downregulating SOX2 expression, and IL-6 can decrease the expression of Zfp637 through the SOCS3/STAT3 signaling pathway.

Zinc finger proteins have been implicated as transcription factors in a variety of pathophysiological processes, including embryo development, hormone secretion, cellular differentiation, senescence, and apoptosis. Zfp637, a transcription factor gene located in the F1 segment of mouse chromosome 6, encodes a zinc finger protein, Mus musculus (GenBank ID: 232337), belonging to the Kruppel-like protein family. Mus musculus comprises six consecutively typical and one atypical C2H2 zinc finger motifs, with 1114 bp of cDNA encoding 272 amino acids. Zfp637 contains protein kinase C phosphorylation sites, phosphoric acid tyrosine kinase phosphorylation sites, N terminal cardamom acylation sites, and epidermal growth factor structural domains^1^. In addition, tissue-specific expression of Zfp637 has been correlated with telomerase activity^1^, and thus, it may be involved in multiple biological behaviors. In our previous studies, we confirmed that Zfp637 could repress myogenic cellular differentiation^2^, but the specific functions of Zfp637 in germ cell differentiation remain unknown. In this study, we examined the expression of Zfp637 in mouse spermatogonia and then evaluated the effects of Zfp637 overexpression or underexpression on germ cell differentiation.

The association of obesity with decreased sexual function and sexual development disorder has been recognized. Obesity in males can manifest as sexual organ hypoplasia and delayed puberty^3^,^4^. Studies investigating sexual development in male children with simple obesity have demonstrated that the testicular volume, size, serum luteinizing hormone, follicle stimulating hormone, and testosterone are reduced compared with the control groups^5^,^6^. Obese males are more likely to suffer from sexual development disorders, but the mechanism by which obesity affects sexual development is still unclear. In our previous research using mouse testis tissue immunohistochemistry experiments, we discovered that the expression of Zfp637 was significantly downregulated in male germ cells of obese compared with normal mice (unpublished results). Therefore, we hypothesized that Zfp637 plays an important role during the process of spermatogenesis and is associated with sexual development disorders in obese individuals.

In recent years, obesity has also been widely accepted as a chronic inflammatory disease^7^,^8^. Inflammatory cytokines can also affect the development and function of testes in obese individuals. IL-6 is a multi-functional pro-inflammatory cytokine that is secreted by adipose cells and macrophages, and it has both inflammatory and anti-inflammatory functions^9^. The effects of IL-6 are related to its concentration in the tissue. High levels of IL-6 cause inflammatory damage and play an important role in the process of inflammation and the immune response to infection and injury. IL-6 also can directly damage the structure of the testicular tissue^10^,^11^. As previously reported, IL-6 can directly inhibit testosterone secretion in Leydig cells^12^,^13^, yet no studies have directly linked IL-6 to disturbances in spermatogenesis. In this study, we exposed mouse spermatogonial cells, GC-1 spg, to high levels of IL-6 *in vitro* and examined the effects on germ cell differentiation.

According to our previous observations, the aim of this study was to clarify the precise functions of the zinc finger protein Zfp637 in testicular germ cell differentiation and the mechanisms by which IL-6 mediates the expression of Zfp637 to modulate spermatogenesis.

## Results

### Spermatogenesis is dependent on constitutive expression of Zfp637

**Figure 1:**
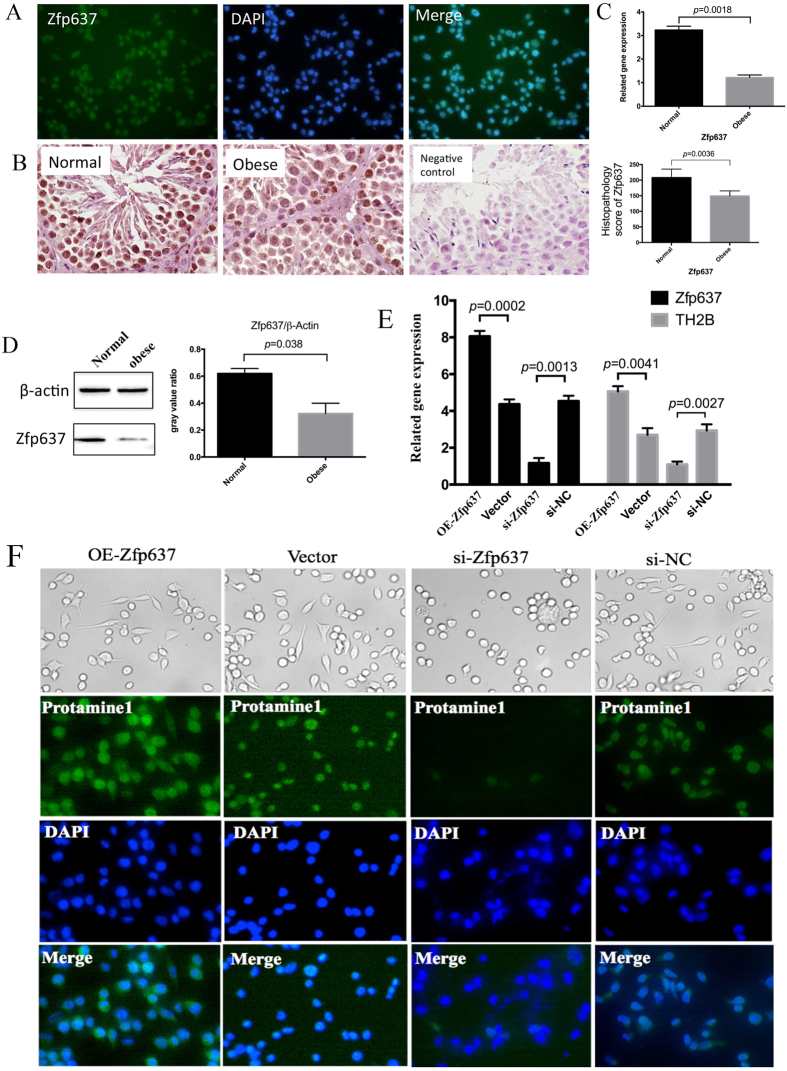
Spermatogenesis is dependent on regular expression of Zfp637. (**A**) ICC showing that Zfp637 is highly expressed and localizes in the nucleus of GC-1 spg cells. (**B**) IHC, (**C**) qRT-PCR and (**D**) western blot analysis were performed to evaluate the expression of Zfp637 in mouse testis tissue. (**E**) Effects of OE-Zfp637 in GC-1 spg cells, Zfp637 expression knockdown by siRNA-Zfp63, and the expression of TH2B detected by qRT-PCR. (**F**) ICC was used to measure the expression of the specific haploid cell marker, protamine 1.

In our previous study, we observed that germ cell counts were reduced in obese compared with control mice, although the precise mechanism underlying this phenomenon remains unclear^13^. According to a bioinformatics analysis, the zinc finger transcription factor protein Zfp637 may play a role in gene regulation, thus impacting the biological behavior of cells. Our previous study has also confirmed that Zfp637 plays an important role in the differentiation of cells^2^. Consequently, immunocytochemistry (ICC) was performed to detect the expression of Zfp637 in mouse spermatogonial GC-1 spg cells. The results revealed that Zfp637 was highly expressed and localized in the nucleus of normal mouse spermatogonial cells (Fig. 1A). This finding suggested that Zfp637 participated in mediating mouse germ cell differentiation. Based on this information, immunohistochemical staining of testicular tissue was implemented to evaluate any differences in the expression of Zfp637 between obese and normal mice. The results verified that Zfp637 was highly expressed in mouse spermatocytes and sperm cells in the testicles of normal mice. However, in the testes of obese mice, the expression of Zfp637 was significantly decreased (Fig. 1B–D). The above results indicate that suppression of Zfp637 plays an important role in the development of obesity-related sexual disorders.

To better understand the relationship between Zfp637 and spermatogenesis, we overexpressed Zfp637 in GC-1 spg cells via transfection with Zfp637 lentivirus plasmid and inhibited Zfp637 expression using siRNA-Zfp63. Next, we cultured the cells in the retinoic acid (RA) culture system to induce differentiation, which is classically used as an inducer *in vitro*. Interestingly, overexpression of Zfp637 promoted cell differentiation and activated the expression of specific haploid cell markers (TH2B and protamine 1) (Fig. 1E,F). In contrast, diminished expression of Zfp637 significantly inhibited cell differentiation (Fig. 1E,F). Based on these results, we concluded that spermatogenesis depended on regular expression of Zfp637.

### Zfp637 may regulate the differentiation of spermatogonia by inhibiting the expression of SOX2

**Figure 2:**
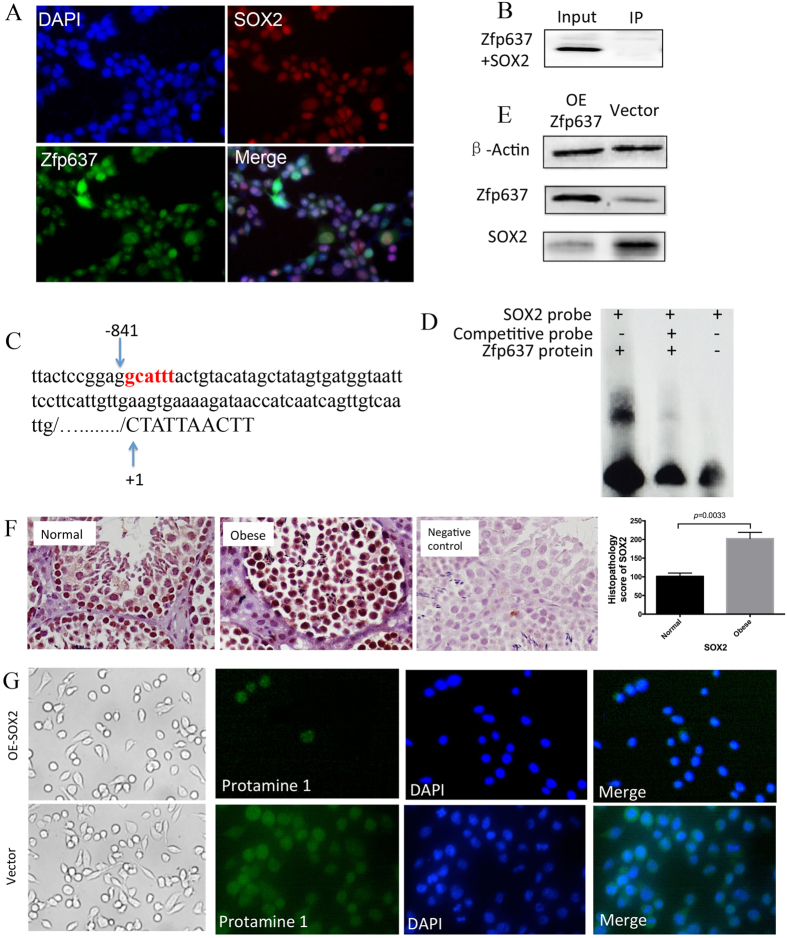
Zfp637 regulates the differentiation of spermatogonia by inhibiting the expression of SOX2. (**A**) ICC showing that Zfp637 and SOX2 are both expressed in GC-1 spg cells and localize in the nucleus. (**B**) Immunoprecipitation showing that Zfp637 and SOX2 protein do not physically interact with each other. (**C**) There is one Zfp637 binding site in the SOX2 promoter. (**D**) EMSA showing the formation of Zfp637 complexes with a probe derived from the SOX2 promoter. (**E**) Western blot showing that the expression of SOX2 is inversely correlated with Zfp637 expression. (**F**) Expression of SOX2 in mouse testicles by IHC. (**G**) Prevention of spermatogonial cell differentiation by overexpression of SOX2.

Zfp637 has been shown to participate in spermatogenesis. However, the mechanism by which Zfp637 regulates the differentiation of spermatogonia requires further investigation. A bioinformatics analysis suggested that Zfp637 might interact with the SOX2 gene. In addition, immunocytochemistry staining confirmed that Zfp637 and SOX2 were both expressed in GC-1 spg cells and localized to the nucleus (Fig. 2A), suggesting their potential interaction. It is well known that SOX2 is located in the SRY gene, which is closely related to male sexual development. Hence, SOX2 is the most likely downstream factor by which Zfp637 regulates spermatogenesis. To verify this hypothesis, immunoprecipitation was performed, but no interaction between Zfp637 and SOX2 protein was detected (Fig. 2B).

Studying these biological results, we discovered one Zfp637 binding site upstream of the SOX2 promoter (Fig. 2C), suggesting that Zfp637, as a transcription factor, can regulate SOX2 expression by interacting with the SOX2 promoter. An electrophoretic mobility shift assay (EMSA) was subsequently performed to verify whether Zfp637 could directly and specifically bind to the SOX2 promoter (Fig. 2D). We demonstrated that Zfp637 formed complexes with a probe derived from the SOX2 promoter. Furthermore, western blot analysis revealed that the expression of SOX2 was negatively correlated with Zfp637 expression (Fig. 2E). In addition, IHC staining revealed that the expression of SOX2 (Fig. 2F) in spermatogonial stem cells was significantly increased in obese individuals. The expression of SOX2 was clearly increasingly accompanied by Zfp637 reduction in obese individuals. To confirm the effects of SOX2 in spermatogonial differentiation, we overexpressed SOX2 in GC-1 spg cells via transfection with a SOX2 lentiviral plasmid. The results showed that increased SOX2 expression was correlated with greater cell proliferation; however, these cells did not enter the stage of differentiation (Fig. 2G). These results confirmed that overexpression of SOX2 could prevent spermatogonial cell differentiation.

### IL-6 can directly abrogate the process of spermatogenesis by inhibiting the expression of Zfp637 in obese individuals

**Figure 3:**
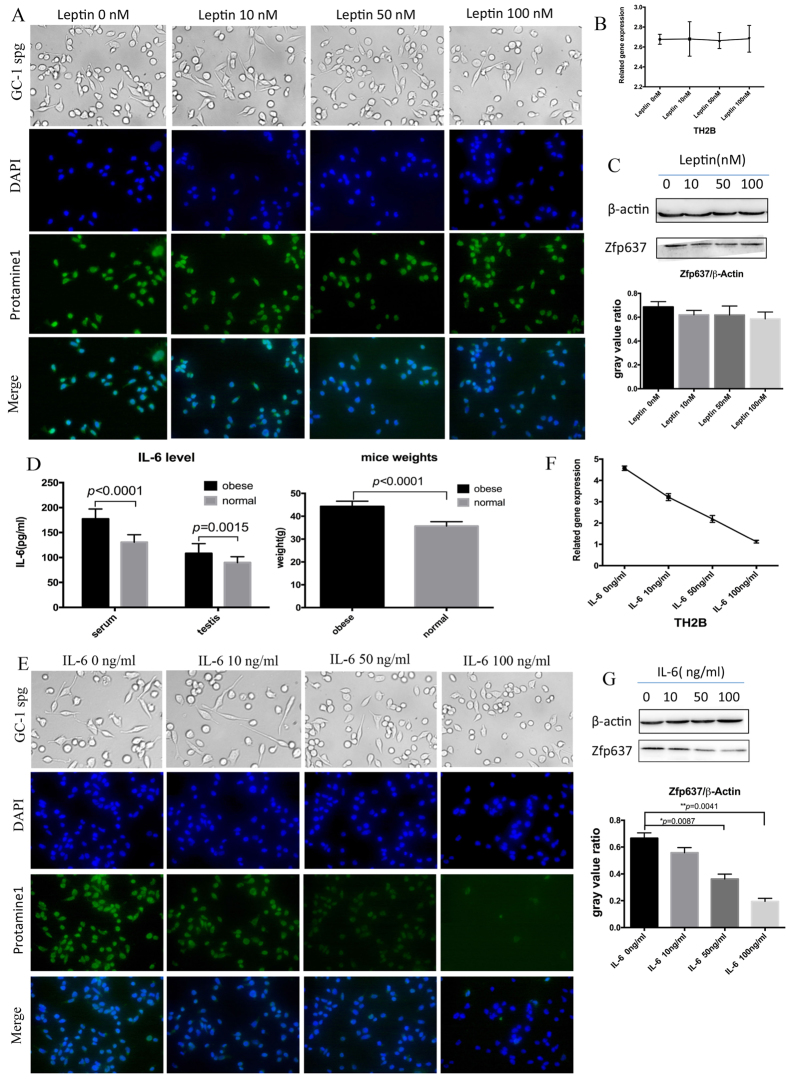
IL-6 can directly disrupt spermatogenesis by inhibiting the expression of Zfp637 in obese individuals. The expressions of protamine 1 (**A**), TH2B (**B**) and Zfp637 (**C**) were measured by ICC, qRT-PCR and western blot, respectively, after a 48-h treatment with Leptin. (**D**) ELISA demonstrating elevated IL-6 levels in obese mice. (**E**) The expression levels of protamine 1, (**F**) TH2B and (**G**) Zfp637 measured by ICC-IF, qRT-PCR and western blot, respectively, after a 24-h treatment with IL-6.

In our previous research^13^, we demonstrated that serum levels of Leptin were elevated and germ cell counts were decreased in obese mice. To determine whether Leptin could directly affect germ cells, we cultivated GC-1 spg cells in the presence of a concentration gradient of Leptin (0, 10, 50 and 100 nM) and then examined the expression of Zfp637 and observed changes in cellular morphology. We observed that even a high concentration of Leptin was unable to suppress the expression of protamine 1 (Fig. 3A), TH2B (Fig. 3B) or Zfp637 (Fig. 3C). These findings indicated that Leptin did not directly influence the differentiation of male germ cells.

**Figure 4:**
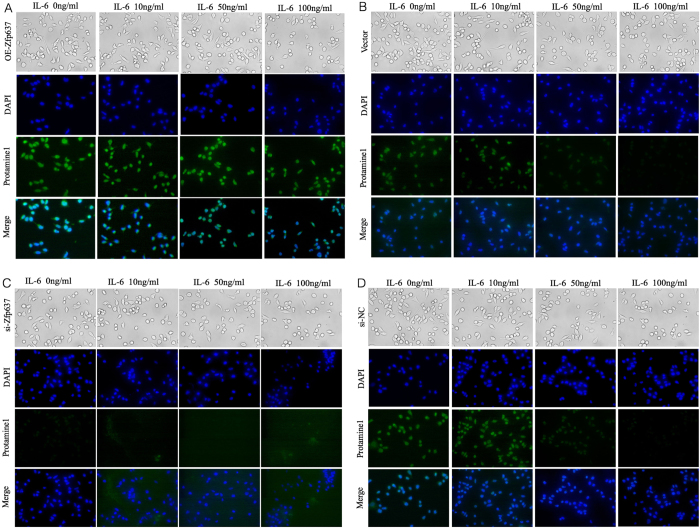
IL-6 can directly disrupt spermatogenesis by inhibiting the expression of Zfp637 in obese individuals. (**A,B**) Cell morphology and expression of protamine 1 in GC-1 spg cells transfected with pcDNA-Zfp637 and pcDNA-Vector, respectively, after a 24-h treatment with IL-6. (**C,D**) Cell morphology and expression of protamine 1 in GC-1 spg cells transfected with siRNA-Zfp637 and si-NC, respectively, after a 24-h treatment with IL-6.

As previously reported, serum levels of IL-6 are elevated in obese males^16^. The presence of IL-6 receptors has been confirmed in male germ cells^17^,^18^. Considering obesity as a chronic inflammatory disease, we examined the levels of IL-6 both in obese and normal mice by ELISA. The obese mice had higher levels of IL-6 both in serum and testis homogenate (Fig. 3.D). Similarly, GC-1 spg cells were stimulated *in vitro* with a concentration gradient of IL-6 (0, 10, 50, and 100 ng/ml) for 24 hours in the previously described culture system. Western blot, ICC and RT-PCR analyses were performed to measure the expression of Zfp637, protamine 1 and TH2B, respectively. Interestingly, high levels of IL-6 clearly inhibited the differentiation of GC-1 spg cells (Fig. 3E,F), as well as the expression of Zfp637 (Fig. 3G). Therefore, we hypothesized that an increase in IL-6 could inhibit the expression of Zfp637, which would further interfere with spermatogenesis. To test this hypothesis, we overexpressed Zfp637 in GC-1 spg cells by transfection with Zfp637 lentivirus plasmid and underexpressed Zfp637 with siRNA-Zfp63. Subsequently, we observed any differences during cell differentiation in response to the concentration gradient of IL-6. Indeed, cells overexpressing Zfp637 could resist IL-6-induced inhibition of spermatogonial differentiation (Fig. 4A,B). Underexpression of Zfp637 significantly enhanced the effect of IL-6 by inhibiting cellular differentiation (Fig. 4C,D). These results demonstrated that IL-6 could alter the process of germ cell differentiation by suppressing the expression of Zfp637.

### IL-6 reduces the expression of Zfp637 via the SOCS3/pSTAT3 signaling pathway

**Figure 5:**
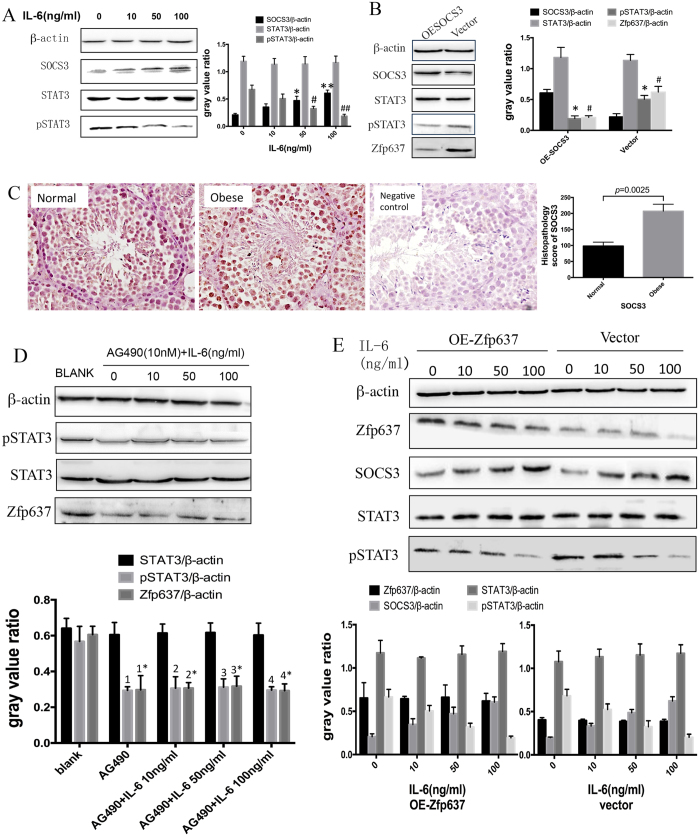
IL-6 reduces the expression of Zfp637 via the SOCS3/pSTAT3 signaling pathway. (**A**) Activity of the SOCS3/STAT3 signaling pathway after exposure of GC-1 spg cells to IL-6 for 24 hours. * and ^#^*p* < 0.01 compared with the IL-6 = 0 ng/ml group. ** and ^##^p < 0.0001 compared with the IL-6 = 0 ng/ml group. (**B**) Expression of STAT3, pSTAT3 and Zfp637 in GC-1 spg cells after OE-SOCS3. *p < 0.001. ^#^*p* < 0.001. (**C**) IHC staining to measure the expression of SOCS3 in normal and obese mouse testes. (**D**) Cells treated with AG490 (10 nM, 2 hours) prior to exposure to IL-6. 1, 2, 3 and 4, *p* < 0.001 compared with the blank group. 1*, 2*, 3* and 4* *p* < 0.001 compared with the blank group. (**E**) Overexpression of Zfp637 did not influence the expression of STAT3, pSTAT3 or SOCS3.

IL-6 and Zfp637 have been shown to be involved in the differentiation of male germ cells. However, the signaling pathway through which IL-6 regulates the expression of Zfp637 requires further investigation. One classic signaling pathway has been implicated, the IL-6/SOCS3/STAT3 signaling pathway, which is a common pathway underlying inflammation and cell differentiation. Suppressor of cytokine signaling 3 (SOCS3), which is an important member of the suppressors of cytokine signaling superfamily, acts as a feedback inhibitor of the JAK–STAT pathway by inhibiting the phosphorylation of STAT3^19^,^20^,^21^. By considering the role of the SOCS3/pSTAT3 signaling pathway in cell differentiation, we hypothesized that IL-6 is likely to regulate Zfp637 expression through this pathway. To investigate this mechanism, western blot analysis was performed to detect the expression of SOCS3, STAT3 and pSTAT3 in GC-1 spg cells after exposure to IL-6 for 24 hours. The results demonstrated that IL-6 could promote the expression of SOCS3. Although the expression of STAT3 was not affected, phosphorylation of STAT3 (pSTAT3) was reduced and was concentration-dependent (Fig. 5A). To further clarify the present hypothesis, we transfected GC-1 spg cells with lentivirus plasmid to overexpress SOCS3. Western blot analysis revealed that in SOCS3-overexpressing cells, the expression levels of both Zfp637 and phosphorylated STAT3 were reduced (Fig. 5B). In addition, IHC revealed increased expression of SOCS3 in obese mouse testis tissue (Fig. 5C). These results further confirmed that the SOCS3/STAT3 signaling pathway could regulate the expression of Zfp637. Subsequently, to determine whether pSTAT3 could influence the expression of Zfp637, we used AG490 (10 nM, 2-hour treatment), a STAT3 phosphorylation inhibitor. The expression of Zfp637 was reduced, even with IL-6 stimulation, when pSTAT3 was blocked (Fig. 5D), demonstrating that pSTAT3 could directly influence the expression of Zfp637. Finally, to assess whether Zfp637 could activate the SOCS3/STAT3 signaling pathway, we exposed GC-1 spg cells to IL-6 for 24 h after overexpression of Zfp637, and then detected the expression of SOCS3, STAT3 and pSTAT3 by western blot analysis. Our results showed that overexpression of Zfp637 did not influence the expression of STAT3, pSTAT3 or SOCS3 (Fig. 5E), suggesting that the regulation of Zfp637 by the SOCS3/STAT3 pathway is unidirectional and is not interdependent. These findings confirmed that IL-6 reduced the expression of Zfp637 through the SOCS3/pSTAT3 signaling pathway.

## Discussion

A number of zinc finger proteins have been shown to have critical roles in cellular proliferation, differentiation, and senescence. Our previous studies have confirmed that the new zinc finger protein, Zfp637, plays an important role in the differentiation of cells^1^,^2^ However, no research has been conducted to specifically determine whether Zfp637 regulates the differentiation of germ cells. In the present study, we confirmed that the expression of Zfp637 was decreased in the testicular tissue of obese mice. Using animal experiments, we also demonstrated that the levels of IL-6 were increased in serum and testis homogenate in obese mice. In addition, *in vitro* experiments revealed that high levels of IL-6 could directly inhibit the differentiation of male mouse germ cells, and overexpression of Zfp637 could prevent the differentiation disruption and the damage to spermatogonia. To the best of our knowledge, this is the first study to evaluate Zfp637 and male germ cell differentiation. Zfp637 has been shown to promote the differentiation of spermatogonia but functions as a repressive regulator in myogenic cellular differentiation^1^,^2^. We speculate that the effect of Zfp637 is dependent on the cell type. Our results provide a reliable scientific basis for the mechanism by which inflammation in obesity affects male sexual development. Although we clearly demonstrated that Zfp637 had an effect on spermatogonial differentiation, these results were obtained in an experimental animal model and have yet to be confirmed in human tissue. In future investigations, we will study the effects of ZNF32, which is a homologous gene of human Zfp637, on male germ cell differentiation and maturation.

It is widely accepted that the IL-6/JAK/STAT3 pathway is involved in inflammation, cellular differentiation, and many other physiological activities of cells. SOCS proteins can inhibit the catalytic activities of enzymes by competing with substrates that play a role further downstream in the signaling pathway. The SH2 domain of SOCS3 binds to the Src homology phosphatase-2 (SHP-2)-binding domains of the gp130 receptor rather than to JAK^22^. It is thought that SOCS3 binds to gp130 receptors and inhibits JAK activity by accessing the activation loop of JAKs via its kinase inhibitor domain^23^. Cytokines are known to induce SOCS3, including the gp130 signaling cytokines (e.g., IL-6, IL-2, IL-3, IL-4, IL-10, type I and type II interferons (IFNs), and Leptin) as well as Toll-like receptor (TLR) agonists (e.g., lipopolysaccharide (LPS), CpG-DNA), growth hormone (GH), prolactin and cyclic AMP-mobilizing hormones^24^. Upon induction, SOCS3 regulates the magnitude, kinetics, and quality of JAK/STAT signaling via multiple receptors. This process is mediated by the binding of SOCS3 to specific PTyr residues of downstream targets via its central SH2 domain^24^. In the present study, we showed that male mouse germ cell expression of SOCS3 is induced by IL-6. The results of the SOCS3 overexpression experiments suggested that the IL-6/SOCS3/STAT3 signaling pathway has a critical role in spermatogenesis. SOCS3 overexpression inhibits STAT3 phosphorylation, as well as STAT3-mediated downstream events.

It remains unknown whether higher levels of serum IL-6 result in traversal of the blood-testis barrier in obese individuals. We were unable to evaluate this phenomenon in the present analysis, which is one limitation of this study. However, IL-6 can affect Sertoli cells, increasing the permeability of the blood-testis barrier^25^. We postulate that serum IL-6 can cross the blood-testis barrier in this manner. In addition, as secretory cells, Sertoli cells and testicular macrophages can secrete IL-6^26^,^27^,^28^. The increased levels of inflammatory cytokines in the microenvironment can induce Sertoli cell to express a large number of inflammatory mediators, which are released into the seminiferous tubule. In the present study, we also detected higher levels of IL-6 in the testis homogenate of obese compared with control mice. Therefore, IL-6 not only influences germ cells directly, which we have confirmed in *in vitro* experiments, but it also alters the differentiation of SSCs by disrupting the blood-testis barrier and damaging the spermatogonial stem cell niche.

As a stem cell factor, SOX2 can inhibit cellular differentiation. SOX2 is located in the SRY gene, which is closely related to male sexual development. Thus, SOX2 might be involved in the regulation of sexual development. Normal expression of SRY is essential for testis development. It is generally believed that male sexual development is more closely related to SOX9 rather than SOX2. Only a handful of studies have investigated the relationship between SOX2 and spermatogenesis, but a recent study has confirmed that SOX2 is expressed in spermatogonia^29^. In the present study, we clearly demonstrated that the expression of SOX2 is inversely related to that of Zfp637 via Zfp637 interactions with the binding site in the upstream promoter of SOX2 in spermatogonial stem cells. However, the mechanism by which spermatogonial differentiation is regulated by SOX2 remains unknown. We hypothesize that SOX2 allows spermatogonial stem cells to maintain their stemness and delays the process of spermatogenesis. In future studies, we will examine how abnormal SOX2 expression specifically affects spermatogenesis.

In conclusion, IL-6 directly regulates the expression of Zfp637 through the SOCS3/STAT3 signaling pathway and thus affects the differentiation of spermatogonia *in vivo* and *in vitro*. Zfp637 can combine with the upstream promoter of the SOX2 gene and downregulates its expression. In obese mice, Zfp637 expression is reduced and the expression of SOX2 is increased. Thus, the spermatogonial stem cells maintain their stem cell status and do undergo differentiation.

## Materials and Methods

### Cell culture and treatment

GC-1 spg cells were obtained from the American Type Culture Collection and maintained in RPMI-1640 medium supplemented with 10% fetal bovine serum, 100 U/ml penicillin, and 100 mg/ml streptomycin. Cells were cultured at 37.1 °C under an atmosphere of 95% humidified air with 5% CO_2_. To induce differentiation, the cells were cultured in medium containing retinoic acid (Sigma Chemical Company, St. Louis, MO, USA) and other growth factors described in literature^14^. To examine the effect of IL-6, the cells were cultured in medium containing 0, 10, 50 and 100 ng/ml IL-6.

### Zfp637 gene silencing

Zfp637 knockdown was performed with specific siRNA targeting Zfp637, which was synthesized by Gene Pharma Company with the following sequences:

Sense: 5′-GUCAGAAAGGAAGCUUAAUdTdT-3′;

Antisense: 5′-UUCUCCGAACGUGUCACGUTT-3′.

### Cell transfection

To obtain cells overexpressing Zfp637 and SOCS3, 1.5 × 10^5^ cells per well in a 6-well plate were transfected with 4 mg of pcDNA3.1-Zfp637 or pcDNA3.1-SOCS3 plasmid using TurboFect (Fermentas, St. Leon-Rot, Germany). Cells overexpressing SOX2 were transfected with SOX2 lentiviral activation particles (Santa Cruz Biotechnology).

### RNA isolation and qRT-PCR

Total RNA was isolated using RNAiso plus (TAKARA, Tokyo, Japan) and converted into cDNA using a PrimeScript RT reagent kit (TAKARA) according to the manufacturer’s instructions. The primer sequences were as follows:

Zfp637: forward, 5′-GCCTTTTTCAATGTGATGACAGA-30′,

Reverse, 5′-TCCCACATTCCTGGCAATC-3′;

TH2B: forward: 5′-CGGTAAAGGGTGCTACTA-3′,

Reverse: 5′-CACTTGTTTCAGCACCTTA-3′;

SOX2 forward, 5′-TGCAGTACAACTCCATGACCAG-3′,

Reverse, 5′-GGGAGGAAGAGGTAACCACAG-3′.

The amplification involved an initial denaturation at 95 °C for 10 seconds followed by 29 cycles of denaturation at 95 °C for 5 seconds, and annealing and extension at 60 °C for 45 seconds. The reactions were performed in triplicate, and the mRNA expression was normalized to the internal 18S control gene.

### ELISA

The levels of IL-6 in mouse serum and testis homogenate were detected using an IL-6 ELISA kit purchased from MeiLian Biotechnology company (MLbio, Shanghai, China), according to the manufacturer’s instructions.

### Western blot analysis

The cells were collected, washed with PBS and then lysed with lysis buffer (50 mM Tris-HCl, 150 mM NaCl, 1 mM EDTA, 50 Mm NaF, 30 mM Na4P2O7, 1 mM phenylmethylsulfonyl fluoride, 2 mg/ml aprotinin) for 30 minutes on ice. After the protein concentrations were determined using the Bio-Rad Protein Assay, equal amounts of extracted protein were separated by 12% SDS-PAGE and then transferred to a polyvinylidene difluoride membrane (Millipore, Bedford, MA, USA). After blocking with TBST (10 mM Tris-HCl, pH 8.0, 150 mM NaCl, 0.1% Tween-20) containing 5% skim milk for 1 h at 37 °C, the membrane was incubated with primary antibody at 4 °C overnight. The antibodies and their sources were as follows: anti-Zfp637 antibody (1:100) was produced and purified as previously described^15^; anti-STAT3 antibody (1:2000) and anti-pSTAT3 (1:1000) antibody were purchased from Cell Signaling Technology (Beverly, MA, USA); anti-β-actin antibody (1:1000) was purchased from Santa Cruz Biotechnology (Santa Cruz, CA, USA); anti-SOCS3 antibody (1:1000) and anti-SOX2 antibody (1:1000) were purchased from Abcam (Abcam, UK). After incubation with the primary antibody, the membrane was washed with TBST and then incubated with horseradish peroxidase-conjugated goat anti-rabbit/mouse antibody (Santa Cruz Biotechnology) for 1 h at room temperature. After washing with TBST, the membrane was developed using Immobilon Western Chemiluminescent horseradish peroxidase Substrate (Merck Millipore, USA).

### Immunocytochemistry

Cells were incubated for 2 hours at 37 °C with goat anti-mouse protamine (1:100 dilution, Santa Cruz Biotechnology), goat anti-mouse SOX2 (1:100 dilution) and rabbit anti-mouse Zfp637 (1:50 dilution) primary antibodies.

### EMSA

Nuclear extracts were prepared using the ProteoJET Cytoplasmic and Nuclear Protein Extraction Kit (Fermentas) according to the manufacturer’s instruction. Nuclear protein was incubated with the following 50-end biotin-labeled double-stranded oligonucleotide: mouse SOX2, 5′-CCATGCCATCAC**GCATTT**TACAGCAACAGGG-3′. The binding reaction was performed for 20 minutes at room temperature using the LightShift Chemiluminescent EMSA Kit (Pierce Biotechnology, Rockford, IL, USA). The protein–DNA complexes were separated by electrophoresis in a 5% non-denaturing polyacrylamide gel and then transferred to a positively charged nylon membrane (Millipore). The membrane was then developed using the Chemiluminescent Nucleic Acid Detection Module (Pierce Biotechnology) according to the manufacturer’s instructions. For the competition assays, the nuclear extracts were incubated with a 200-fold molar excess of unlabeled double-stranded competitor oligonucleotides.

### Tissue dissection from mice

Male BALB/C mice were purchased from the Experimental Animal Center of Sichuan University (Chengdu, China). The high fat diet was purchased from Dashuo Biotechnology Company (Chengdu, China). Each experimental group consisted of six mice. To obtain tissues, mice were sacrificed for blood collection and testicular tissue dissection. All experimental protocols were approved by the Committee on the Use of Live Animals in Teaching and Research of Sichuan University. The procedures were conducted in accordance with approved guidelines.

### Immunohistochemistry

The testes of the mice in each group were isolated and, to maintain integrity, fixed in 4% paraformaldehyde for 48 hours and embedded in paraffin. They were then sliced into 5-μm sections of the testis along the long diameter of the block. The testes were incubated overnight at 4 °C with rabbit anti-mouse SOCS3 (1:400 dilution), goat anti-mouse SOX2 (1:400 dilution) and rabbit anti-mouse Zfp637 (1:100 dilution) primary antibodies. Subsequently, the tissues were incubated with biotinylated goat anti-rabbit (1:800 dilution, Zhongshanjinqiao, China) and biotinylated mouse anti-goat (1:800 dilution, Zhongshanjinqiao, China) immunoglobulin secondary antibodies. The slides were then counterstained with hematoxylin. A goat isotype IgG (1:400 dilution, Zhongshanjinqiao, China) was served as the corresponding native control.

### Statistical analysis

All quantitative data are expressed as the means ± S.D. To determine significance between two groups, comparisons between means were performed using Student’s t-test. To compare all groups, one-way ANOVA was used.

## Additional Information

**How to cite this article**: Huang, G. *et al*. IL-6 mediates differentiation disorder during spermatogenesis in obesity-associated inflammation by affecting the expression of Zfp637 through the SOCS3/STAT3 pathway. *Sci. Rep.* **6**, 28012; doi: 10.1038/srep28012 (2016).
